# The Clinical and Safety Outcomes of 27 Gauge Pars Plana Vitrectomy in Eyes With Macular Hole

**DOI:** 10.7759/cureus.24409

**Published:** 2022-04-23

**Authors:** Muhammad Amer Awan, Fiza Shaheen, Aqdus Haq, Sahar Fatima

**Affiliations:** 1 Ophthalmology, Shifa International Hospital, Islamabad, PAK; 2 Ophthalmology, Shifa Tameer-E-Millat University, Shifa College of Medicine, Islamabad, PAK

**Keywords:** vitreoretinal surgery, vitrectomy, visual acuity, retina, eye

## Abstract

Objective

To report the clinical, visual, and safety outcomes of 27 gauge pars plana vitrectomy (27G PPV) in eyes with full thickness and lamellar macular hole (MH).

Methodology

This retrospective cross-sectional study, conducted at the ophthalmology department of Shifa International Hospital, Islamabad, was done on 89 eyes of 85 patients who underwent 27G PPV for full-thickness and lamellar MH with a postoperative follow-up period of six months.

Results

The mean age was 61.9 ± 17.3 years with 57.3% being males. Most of the eyes had idiopathic stage III full-thickness MH (n=34, 38.2 %). The total mean operating time was 42.5 ± 19.8 minutes. During surgery, 48 (53.9%) eyes had concurrent phacoemulsification. Hexafluoroethane was the most commonly used tamponade agent (n=81, 91%). Postoperatively, the primary closure rate was 93.2% (n=83) while the final closure rate was 100% (n=6) either by prolonged posturing (n=3, 3.3%) or by an additional procedure involving autologous internal limiting membrane (ILM) transplant (n=3, 3.3%). The complication rate was 2.2% including iatrogenic retinal tear (n=1, 1.1%) and raised IOP (n=1, 1.1%). The overall best-corrected visual acuity (BCVA) improved significantly from 1.20 ± 0.67 to 0.31 ±0.17 (p=<0.001).

Conclusion

As per this study, 27G PPV is a practical and efficient surgical system with substantial anatomical success, minimal complication, and considerable visual recovery rates in eyes with full thickness and lamellar MH. We suggest 27G PPV with ILM peeling and medium-acting intraocular gas as the standard procedure for MH.

## Introduction

Trans-conjunctival micro-incisional vitrectomy surgery (MIVS) is one of the most promising and continuously evolving vitreoretinal surgical procedures used for multiple posterior segment diseases [1]. Conventional methods utilize 20, 23, and 25 gauge cutters for this purpose [2]. After the introduction of the 27 gauge cutter by Oshima et al. [3], measuring 0.40 mm with the advantage of a smaller wound, faster healing, and decreased downtime for the patient, traditional approaches using larger gauge cutters are being used to a lesser extent. Although it promises lesser complications such as scleral astigmatism, and post-operative infection with earlier visual recovery [4,5], some studies have shown concern for instrument friability and wound leak [6,7]. 

The range of pathologies for which the 27 gauge system can be used is expanding day by day and with increasing surgeon’s confidence, surgical outcomes are being presented for a variety of vitreoretinal (VR) diseases from simple to complex variety. In literature, a lot of work has been done on comparison between 25 gauge and 27 gauge systems [8-10] for a variety of indications and comparable outcomes have been reported. Despite its widespread use, currently, less data is available that categorically establishes the use of 27 gauge pars plana vitrectomy (27G PPV) in the treatment of macular holes (MH). In this study, we aim to investigate the clinical, visual, and safety outcomes for patients undergoing MIVS using a 27 gauge system for MH.

## Materials and methods

After gaining approval from the Institutional Review Board (IRB) and Ethics Committee (Reference # 219-709-2019), this single-center retrospective, cross-sectional study was conducted at the ophthalmology department of Shifa International Hospital, Islamabad. Due to the retrospective nature of this study, informed consent was not obtained from the subjects. Medical records of all the eyes that underwent 27G PPV for MH from July 2017 to June 2020 were analyzed. MH was classified based on the standard classification proposed by the International Vitreomacular Traction Study group by utilizing optical coherence tomography (OCT) scan [11].

All the eyes with MH having pre-operative and post-operative recorded OCT scans who underwent 27G PPV by a single VR surgeon having a postoperative follow-up period of at least six months were included in our study. Eyes with non-availability of OCT scans and with a lack of recorded postoperative data till six months were excluded from our study. Recorded data variables include; age, gender, laterality, stage of MH, etiology, pre-operative and post-operative best-corrected visual acuity (BCVA), and postoperative complications with their management. Surgical data were also collected including date of surgery, duration of surgery, type of anesthesia, surgical steps, use of laser/cryotherapy, and type of tamponade. Post-operative data were collected for up to six months and any additional procedure or advice was also noted. Post-operative intraocular pressure (IOP) of 5 mmHg or less on the Goldmann tonometer was taken as hypotony while an IOP of more than 21 mmHg was noted to be high. 

Primary closure was defined as complete closure of MH after the initial surgery as demonstrated by an OCT scan after the first postoperative week. Partial closure was defined when a defect was not completely closed but there was an improvement in comparison with a pre-operative OCT scan. Failure of closure or no closure was taken when there was a persistent defect with no improvement in pre and post-operative OCT scans. Final closure was defined as closure of MH either by additional posturing or a second procedure. 

Surgical procedure

General anesthesia or modified retrobulbar block (1% lignocaine with 0.5% bupivacaine in 50:50 ratio) given through the inferior fornix at the junction of medial 2/3rd and lateral 1/3rd using a 25 gauge needle was used before surgery for patient’s comfort. Eyes with visually significant cataracts underwent cataract extraction via phacoemulsification before MH surgery. A nearly consistent surgical approach was adopted in all the eyes for MH surgery. However, slightly different maneuvers were done in some eyes depending on the etiology and complications encountered. All the surgeries were performed by a single VR surgeon (MAA).

For PPV, the 27G+ Constellation Vitrectomy System (Alcon Laboratories, Fort Worth, TX) was employed using an angled approach of trocar cannulas. Fundus viewing was done via a non-contact wide-angle viewing system (BIOM, Oculus Inc., Wetzlar, Germany) while a magnification contact lens was also occasionally used in eyes with limited view. After core vitrectomy (7500-10,000 cuts per minute) posterior vitreous detachment (PVD) was induced in all eyes with a vitrectomy probe by aspiration and mechanical uplifting of vitreous in the peri-papillary area in eyes without PVD. Triamcinolone acetonide (0.2 mL of 40mg/mL in 0.8 mL balanced salt solution) was also used in a few eyes for enhanced visualization to induce PVD, thorough and complete vitreous removal.

In eyes with concurrent epiretinal membrane (ERM), peeling was done after instilling Membrane-Blue-Dual dye (DORC International, The Netherlands) twice for one minute followed by ERM peeling and inner limiting membrane (ILM) peeling if the area was stained the second time. While in eyes where just ILM peeling was required, ILM-BLUE dye (DORC International) was used followed by ILM peeling using the maculorrhexis maneuver. For peeling, 27 gauge forceps were employed. In eyes with persistent open holes after the failed primary procedure and extensive posturing trial, an autologous ILM transplant was performed. A patch of ILM from a stained area outside the previously peeled area was grasped and inserted to cover the MH. Retinal breaks were repaired using an endolaser or cryotherapy. Fluid air exchange was done followed by a tamponade agent using air, sulfur hexafluoride (S6), hexafluoroethane (C2F6), or perfluoropropane (C3F8). Closure of the sclerotomy site was ensured with gentle massaging. None of the eyes required suturing of wounds. None of the maneuvers required conversion to a 25- or 23-gauge system. Surgery was concluded with a subconjunctival antibiotic (gentamycin) and corticosteroid (dexamethasone) injection. 

Post-operatively, all the eyes underwent a complete ophthalmic examination on Day 1 then one week and one month after surgery during which slit-lamp examination and Goldmann applanation tonometry was done. Facedown posturing (FDP) was advised for four to six hours to all the subjects for a day with at least 50 minutes per hour. FDP for one week was advised to the eyes with partial or no closure as assessed by OCT scan on Day 1 or after one-week post-op. BCVA was assessed after complete gas resorption using the Snellen visual acuity chart. OCT was done on post-op Day 1, after one week, and again after gas resorption to ascertain the degree of MH closure. 

Statistical analysis

Data were analyzed using SPSS version 21.0 (IBM Corp., Armonk, NY). For qualitative measures, frequency and percentage were used and for quantitative measures, the mean and standard deviation was used. One sample t-test was used for BCVA while an independent t-test was used for duration comparison between phacovitrectomy and vitrectomy. Snellen visual acuity was converted into a minimum angle of resolution equivalents (logMAR) for statistical calculations. Counting fingers and hand movement vision were changed to 1.98 and 2.28 respectively as explained in previous literature [12]. A p-value of <0.05 was considered to be statistically significant.

## Results

Of 85 patients, 89 eyes met our inclusion criteria and were included in this study. The mean age was 61.9 ± 17.3 years (median, 66; range, 11-93 years). Around 57.3% (51) were males and there was 44.9% involvement of the left eye in our patients. Most of the eyes had idiopathic stage III MH (n=34, 38.2%). Stages and etiologies are listed (Table 1).

**Table 1:** Types of macular hole Different types of macular holes and their percentages. MH = macular hole

Stage and etiology	Number (Percentage)
Stage II idiopathic MH	3 (3.4%)
Stage III idiopathic MH	34 (38.2%)
Stage IV idiopathic MH	32 (36%)
Traumatic MH	9 (10%)
Lamellar MH	11 (12.4%)

Pre-operatively, modified retro-bulbar anesthesia was utilized in 85 (95.5%) eyes while four (4.5%) eyes required GA due to younger age or patient’s preference. None of the patients had anesthesia-related complications.

Per-operatively 48 (53.9%) eyes had concurrent phacoemulsification with intraocular lens implantation before vitrectomy. Other significant surgical maneuvers carried out intraoperatively included ILM peel (n=80), ERM peel (n=9), and ILM peel accompanied by an ILM patch as a part of a secondary procedure (n=3). Among the per-operative tamponades; C2F6 was the most commonly used tamponade (n=81, 91%), while air (n=3, 3.4%), C3F8 (n=2, 2.25%) and SF6 (n=2, 2.25%) were also used. None of the surgeries required conversion to larger gauge instrumentation during any step. None of the eyes required suturing of the sclerotomy site towards the end. A detail of tamponades along with the stage and type of hole is given in Table 2.

**Table 2:** Types of macular holes and tamponade agents used. Types of tamponade agents and macular holes. MH = macular hole

Per-operative Tamponade	Stage and Type of macular hole (MH)
Air	Lamellar MH (n=3)
SF6	Stage II (n=1)
SF6	Stage IV (n=1)
C2F6	Stage II (n=2)
C2F6	Stage III (n=34)
C2F6	Stage IV (n=29)
C2F6	Traumatic MH (n=9)
C2F6	Lamellar MH (n=7)
C3F8	Stage IV (n=2)

The total mean operating time was 41.1 ± 17.1 minutes (range: 16-91 mins) with the time difference between combined phaco-vitrectomy and vitrectomy alone (p=0.5) using an independent sample t-test. Data of the patients were followed for six months and outcomes were noted. Complete anatomical closure of MH including lamellar MH was achieved in 83 eyes (93.2%) whereas partial closure was achieved in three eyes (3.3%) and no closure in three eyes (3.3%) after the primary procedure. Eyes with partial closure were advised strict positioning for one week resulting in its closure. Eyes with persistent open MH (n=3, 3.3%) underwent a second procedure comprising of ILM peeling and autologous ILM transplant with C2F6 gas tamponade. All of these eyes achieved closure of MH with a secondary procedure. Our results thus show a primary closure rate of 93.2% and a final closure rate of 100%. Among complications, one eye had an iatrogenic retinal tear (1.1%) which was sealed per-operatively while another eye developed raised IOP (1.1%) in the early postoperative period which was managed with anti-glaucoma drops.

**Figure 1:**
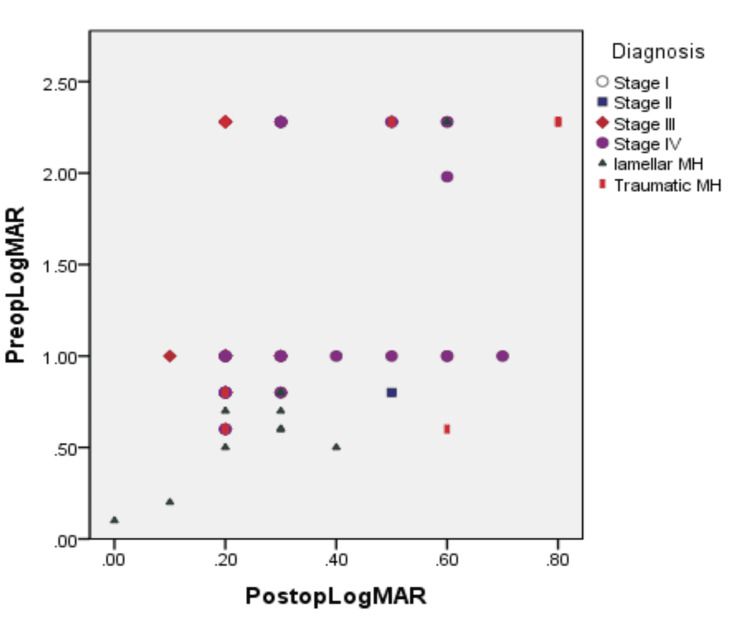
Visual outcome in different types of macular holes. It shows preoperative and postoperative visual acuity in different types of macular holes. MH = macular hole

Overall BCVA improved significantly from 1.20 ± 0.67 to 0.31 ±0.17 (p=<0.001). For stage II, BCVA improved from 0.8 ± 0.2 to 0.3 ± 0.15 (p=0.06). For stage III, BCVA improved from 1.1 ± 0.5 to 0.2 ± 0.07 (p<0.001). For stage IV, BCVA improved from 1.3 ± 0.65 to 0.34 ± 0.16 (p<0.05). For traumatic MH, BCVA improved from 1.7 ± 0.8 to 0.54 ± 0.27 (p<0.001). For lamellar MH, BCVA improved from 0.68 ± 0.56 to 0.27 ± 0.15 (p<0.001). BCVA for each diagnosis is also given in the scatter plot (Figure 1).

## Discussion

MH is a defect in the macula that can be caused by age, trauma, or vitreoretinal disease resulting in impaired central vision, metamorphopsia, and central scotoma [13]. Currently, the standard treatment of choice for MH is vitrectomy with posterior hyaloid face (PHF) removal with ILM peeling with tamponade agent but posturing is still a debatable topic [14]. Vitrectomy can be performed using different gauge cutters. However, with the introduction of MIVS, smaller gauge instrumentation is being exceedingly brought into practice considering its safety profile. However, as with every other new surgical advancement, there are some concerns of complications with this system as well. In our study, we have retrospectively analyzed the surgical outcomes of MH in 89 eyes using MIVS with 27 gauge instrumentation using a more or less similar surgical approach.

The mean age in our data is 61.9 ± 17.31 (11-93) years which is almost similar to reported in the literature [13,15] indicating no significant age difference in this pathology in the Asian population. The total mean operating time reported in our data is slightly longer compared with the 25-gauge system [15], a finding reported before as well [4]. This slightly increased duration has been attributed previously to the low flow of the 27-gauge system owing to the shorter internal diameter of the probe and longer length of the tube [11]. However, with further advancements such as an increase in cut-rate from 10,000 to 20,000, the duration of surgery is further reduced. We also attribute this long time to a greater percentage of concurrent phacoemulsification in our data.

We obtained a nearly uniform surgical approach with slight changes. ILM peeling was done in all the eyes undergoing the first surgery. Although the initial procedure for MH surgery described by Kelly and Wendel [16] involved only vitrectomy with PHF removal to alleviate traction, ILM peeling was introduced later on to further reduce the tangential traction and induce myofibroblast growth that further aids closure [17]. A lot of comparative studies have been done in literature with higher closure rates in eyes undergoing ILM peeling [18,19], an approach supportive of our results as well. An autologous ILM transplant was performed in three eyes in our study with refractory MH using the approach described by Morizane et al. [20] who have reported a success rate of 90% in refractory MH. All of these three eyes achieved anatomical closure along with improved BCVA. We used intermediate-acting intraocular gas as a tamponade agent in most of the eyes owing to its greater success rate in literature (97%) [21] and earlier rehabilitation of vision. Even in failed MH, silicone oil was not used owing to its poorer visual outcomes [22] and the need for a second procedure along with a higher rate of complications. However, in lamellar MH, air was used and found to be a sufficient tamponade agent. Although FDP and its duration is still a debatable topic, we advised posturing for 4-6 hours immediately after surgery. However, if there was no closure of MH on Day 1 OCT or very large MH (greater than 800 microns), patients were advised to posture four to six hours a day for one week. Guillaubey et al. [23] reported 95.1 and 79.5% primary closure rates in postured and non-postured eyes respectively in a prospective RCT. Yorston et al. [24] did not find any difference between posturing and non-posturing patients with MH. Studies claiming little or no importance of FDP are limited in the sample size [25, 26]. Although it is known to cause difficulty in the subjects [27], we suggest that proper counseling and explaining its benefit on anatomic and visual outcomes may make it tolerable to the patient.

The primary success rate in our study is 93.2% which is comparable to 93.3% reported with the use of 23- and 25-gauge systems [28]. Yoneda et al. [29] and Saleh et al. [1] have previously reported a 100% primary closure rate with a 27-gauge system; however, their data is limited by its smaller sample size for MH (n=26 and n=4 respectively). The final closure rate in our data either by an extended FDP of one week or after another surgery is 100%. None of the studies have reported final closure rates yet. However, closure of refractory MH with autologous ILM transplant has been reported to be 90% [21]. Table 3 comprises of characteristics of some of the previous studies given.

**Table 3:** Previous studies compared to the present study This table compares previous studies to the present study. PHF = posterior hyaloid face; MH = macular hole, RPE = retinal pigment epithelium, ILM = inner limiting membrane, SF6 = sulfur hexafluoride, FDP = face down posture, C3F8 = perfluoropropane, RRD = rhegmatogenous retinal detachment, ERM = piretinal membrane.

Study	System	Sample size	Technique	Duration of surgery in minutes	Primary closure rate	Secondary closure rate	Complications	BCVA Improvement
Kelly and Wendel, 1991 [16]	20G	52	PHF removal		58%		15.3%; increased size of MH (3.8%), mottling of RPE (5.7%), vein occlusion (1.9%)	73% had improved
Yoneda et al., 2017 [29]	27G	26	ILM peel, SF6 Gas	34.6± 9.4	100%			Improved p- <0.001
Stene-Johansen et al., 2019 [28]	23/25G	198	ILM peel, SF6 Gas, limited FDP		93.3%		42.4%; retinal detachment (0.5%), VH (2.5%), incomplete PVD (1.5%), lens damage (0.5%), retinal break (12.1%), raised IOP (2.5%), cystoid macular edema (1%), cataract (23 cases)	Improved
Dikci et al., 2019 [12]	23G	17	ILM peel, SF6/C3F8 gas		76.5%		Raised IOP (17.6%), RRD (11.8%), cataract (1.6%),	Improved
Brown et al., 2020 [14]	25G, 27G	47, 10	ILM peel, gas	32.5, 36.7	97.8%, 80%		Retinal tears 4:1 (25g:27g)	Improved P<0.05
Present study	27G	89	ERM, ILM peel, gas/air, FDP	42.5 ± 19.8	93.2%	100%	2.2%; retinal tear (1.1%), raised IOP (1.1%)	Improved P<0.01

We have reported a 2.2% risk of complications in our data, the lowest percentage reported so far. None of the eyes developed sclerotomy site leakage, post-operative early or late hypotony, and endophthalmitis, a major concern previously [5-7]. None of the eyes in our study developed RRD as reported previously [13,29] so we recommend meticulous complete vitreous shaving with scleral depression. Mean BCVA has significantly improved in our study as reported previously as well [15,1]. 

In literature, most studies have analyzed the outcomes in comparison to 25-gauge systems and they are limited by their smaller sample size with the inclusion of only idiopathic MH. While in our study, the sample size is relatively larger, we have also included traumatic MH, lamellar MH caused by central retinal vein occlusion, uveitis, and ERM. The limitations of our study include its retrospective study design, only six months follow-up, and the absence of a control group. Our study did not take into consideration the duration of MH, OCT features including hole height, basal diameters, and choroidal thickening. Our follow-up was limited to six months whereas literature shows that visual acuity keeps improving till 12 months post-operatively. A study with a clinical trial design and a longer follow-up is required to answer these questions.

## Conclusions

In conclusion, 27G PPV is a practical and efficient surgical system with substantial anatomical success, minimal complication, and considerable visual recovery rates in eyes with primary or secondary MH. We suggest 27G PPV with ILM peeling and medium-acting intraocular gas as the standard procedure for MH.
